# Reduced CSF orexin levels in rats and patients with systemic inflammation: a preliminary study

**DOI:** 10.1186/s13104-022-06121-0

**Published:** 2022-06-25

**Authors:** Yasuhiro Ogawa, Nobutake Shimojo, Akiko Ishii, Akira Tamaoka, Satoru Kawano, Yoshiaki Inoue

**Affiliations:** 1 Department of Internal Medicine, University of Tsukuba Hospital, Tsukuba, Ibaraki Japan; 2 Department of Emergency and Critical Care Medicine, Faculty of Medicine, University of Tsukuba, Tsukuba, Ibaraki Japan; 3 Department of Neurology, Faculty of Medicine, University of Tsukuba, Tsukuba, Ibaraki Japan

**Keywords:** Orexin, Cerebrospinal fluid, Systemic inflammation, Sepsis

## Abstract

**Objective:**

Sepsis is a lethal condition characterized by systemic inflammation and multiple organ failure; this condition was initially defined as systemic inflammatory response syndrome (SIRS) due to infection. We previously reported that the hypothalamic neuropeptide orexin improved survival in a murine model of sepsis by mainly acting in the medullary raphe nucleus through orexin type-2 receptors. We hypothesized that orexin treatment enhances recovery from sepsis by reversing the reduction in orexin levels in the cerebrospinal fluid (CSF). We recently reported a case in which CSF orexin levels were reduced in a patient with sepsis. Herein, we attempted to further investigate CSF orexin levels in rats and patients with systemic inflammation. This patient study was a single-center, retrospective observational study.

**Results:**

CSF orexin levels were low in rats with lipopolysaccharide-induced systemic inflammation. We enrolled 14 patients with meningitis/encephalitis. Six patients were diagnosed with SIRS, of whom 5 patients had infections (“sepsis” by the previous definition). CSF orexin levels were low in SIRS patients. The results support the hypothesis that orexin treatment enhances recovery from sepsis by reversing the reduction in CSF orexin levels.

**Supplementary Information:**

The online version contains supplementary material available at 10.1186/s13104-022-06121-0.

## Introduction

Sepsis was initially defined as systemic inflammatory response syndrome (SIRS) due to infection [1, 2]; the definition was recently changed to life-threatening organ dysfunction caused by a dysregulated host response to infection [3]. The brain may be suitable as a novel therapeutic target for sepsis because sepsis-associated encephalopathy is an independent risk factor for mortality in patients with sepsis [4].

Orexin is a neuropeptide produced in the lateral hypothalamus; the secretion of this substance from orexin-producing neurons regulates the sleep/wake cycle through its regulatory roles in the autonomic nervous system, neuroendocrine system, and monoaminergic system [5]. In healthy humans, orexin can be detected (range: 250–500 pg/ml) in cerebrospinal fluid (CSF), and its concentration shows a circadian rhythm. Orexin deficiency causes narcolepsy, a sleep disorder, and CSF orexin levels are 100 pg/ml or less in patients with type 1 narcolepsy. In patients with certain pathological conditions of the brain (e.g., head trauma and Guillain–Barre syndrome), CSF orexin levels are moderately low (range 100–200 pg/ml). However, orexin is generally not detected (< 40 pg/ml) in blood by a standard orexin radioimmunoassay, which is used for the diagnosis of type 1 narcolepsy [6]. An enzyme-linked immunosorbent assay (ELISA) for orexin is available and may be easier to perform than a radioimmunoassay. However, orexin levels measured by ELISA do not match those measured by radioimmunoassay, and there is a clinical discrepancy. Therefore, orexin-based ELISA is not a standard method for the diagnosis of type 1 narcolepsy [7]. Indeed, narcolepsy has been misdiagnosed by CSF orexin levels measured by ELISA [8]. CSF orexin levels in rats are slightly higher than those in humans and are high during the dark (active) period but decrease by 40% toward the end of the light (rest) period [9]. Orexin deficiency induces narcolepsy-like symptoms in rats, similar to patients with narcolepsy [10].

We previously reported that orexin improved survival in mice with endotoxin shock, a well-studied model of sepsis, by mainly acting in the medullary raphe nucleus through orexin type-2 receptors [11]. Regarding the principle of this therapeutic effect, we hypothesized that reduced CSF orexin under the condition of systemic inflammation is restored by orexin treatment. The results obtained from animal experiments need to be carefully validated in patients with sepsis. We recently reported a case in which CSF orexin levels were reduced in a patient with sepsis. Reduced CSF orexin levels returned to normal after the patient recovered from sepsis. Unexpectedly, orexin was detected in the blood, suggesting that CSF orexin might leak into the blood because of blood–brain barrier dysfunction. As a result, the abnormal detection of blood orexin could predict reduced CSF orexin levels [12]. In this study, we first investigated orexin levels in the CSF and blood of rats with lipopolysaccharide-induced systemic inflammation. Next, we attempted to validate orexin levels in the CSF and blood in patients with sepsis. However, it is not ethically acceptable in Japan to perform lumbar puncture in patients with sepsis. Instead, we retrospectively selected patients who were diagnosed with SIRS among the patients who were admitted to the Department of Neurology, where lumbar puncture is routinely performed during hospitalization. This patient study was a single-center, retrospective observational study. To the best of our knowledge, no previous studies have measured CSF orexin levels in patients with sepsis and compared the orexin levels in CSF with those in blood under the condition of systemic inflammation.

## Main text

### Methods

#### Animal experiments

Animal experiments were performed by Charles River Laboratories Japan, Inc. Rats (Crl:CD(SD), 8 weeks old, male) were individually housed and kept on a 12 h:12 h light:dark cycle at an ambient temperature of 23 ± 1 °C under specific-pathogen-free conditions. To induce systemic inflammation, we intraperitoneally injected lipopolysaccharide (LPS, derived from *Escherichia coli* 055:B5; Sigma; 1 mg/kg) into rats at zeitgeber time (ZT) 10.5. At 13.5 h after LPS injection (ZT0), we assessed systemic inflammation as manifested by hypothermia (< 36.0 °C) and weight loss (Additional file 1: Table S1), and we sampled cerebrospinal fluid (CSF) and blood from the rats under anesthesia. As a control, we injected saline into rats at ZT10.5. At 13.5 h after saline injection, animals that showed normothermia and no weight loss were assigned to the control group (Additional file 1: Table S1), and we sampled CSF and blood from them under anesthesia. All the animals were anesthetized with 4% isoflurane during sampling and were euthanized by phlebotomy under anesthesia after sampling.

#### Patient study

In total, 1051 CSF samples and 1164 plasma samples were stored in the Department of Neurology, University of Tsukuba Hospital, from Jan 1, 2013, to Dec 31, 2018. Among them, 220 pairs of CSF and plasma samples were collected on the day of admission, among which 22 pairs were sampled from patients with meningitis/encephalitis. Ultimately, we enrolled 14 patients with meningitis/encephalitis because the CSF and plasma samples of 8 patients had already been used and were not available (Additional file 2: Figure S1). Among the 14 enrolled patients, we retrospectively diagnosed 6 patients with SIRS according to the SIRS criteria (1. body temperature > 38.0 °C or < 36.0 °C, 2. heart rate > 90 beats/minute, 3. respiratory rate > 20 breaths/minute, and 4. white blood cell count > 12 × 10^3^/μl or < 4 × 10^3^/μl).

#### Orexin measurement

Orexin levels were measured using a standard orexin radioimmunoassay (Phoenix Pharmaceuticals, Burlingame, CA, USA) at the International Institute for Integrated Sleep Medicine (WPI-IIIS), University of Tsukuba; this center measures orexin levels continuously to diagnose narcolepsy. The detailed measurement method has previously been reported [13].

### Statistical analysis

We used PRISM Ver. 5.0 for statistical analysis. To compare the averages of continuous values between the 2 groups when the assumption of a Gaussian distribution was met, we performed a t test or Welch’s test following the F test. When the assumption of a Gaussian distribution was not met, we compared the averages of continuous values between the 2 groups using a Mann–Whitney U test. To compare the ratios of categorical values between the 2 groups, we performed Fisher's exact test.

### Results

#### Orexin levels in rats with systemic inflammation

**Fig. 1:**
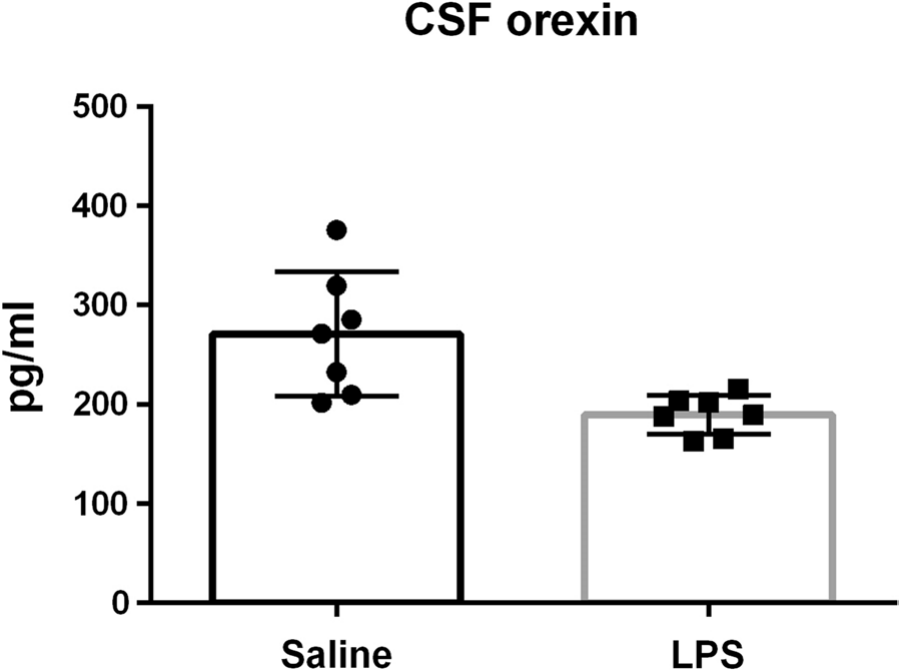
CSF orexin levels in rats with LPS-induced systemic inflammation were significantly lower than those in saline-injected rats (Saline: n = 7, 271.2 ± 57.9, LPS: n = 7, 190.1 ± 18.1, F test: F = 10.2330, p = 0.0123, Welch’s test: p = 0.0131). Data are represented as the mean ± SD. *CSF* cerebrospinal fluid, *LPS* lipopolysaccharide, *SD* standard deviation

We induced systemic inflammation in rats by intraperitoneally injecting lipopolysaccharide (LPS, 1 mg/kg). As a control, we injected saline instead of LPS. At 13.5 h after injection, we assessed systemic inflammation based on physical signs and sampled cerebrospinal fluid (CSF) and blood (Additional file 1: Table S1). CSF orexin levels were significantly lower in rats with LPS-induced systemic inflammation than in saline-injected rats (Fig. 1). Blood orexin was detected in some rats, and CSF orexin levels tended to be reduced in rats with blood orexin detected (Additional file 1: Table S1), but there were no significant differences in CSF orexin levels between rats with blood orexin detected and rats with blood orexin undetected.

#### Clinical data and orexin levels in patients with SIRS

We enrolled 14 meningitis/encephalitis patients whose CSF and blood were sampled on the day of admission to the Department of Neurology, University of Tsukuba Hospital, from Jan 1, 2013, to Dec 31, 2018 (Additional file 2: Fig. S1). We considered the collection times of the blood and CSF samples because CSF orexin fluctuates in a circadian rhythm. The samples were collected between 11:45 AM and 8:00 PM. Based on the SIRS criteria, 6 patients were assigned to the SIRS group, of whom 5 patients had infection (“sepsis” by the previous definition). Quick Sequential Organ Failure Assessment (qSOFA) scores, which have been used as a primary screening tool for sepsis under its new definition, were calculated; only 3 patients had abnormal qSOFA scores (Additional file 3: Table S2). Using blood tests, we could not find evidence of liver injury, kidney injury, or coagulopathy between the SIRS and non-SIRS groups (Additional file 4: Table S3). The results indicated that the incidence of multiple organ failure in the enrolled patients was relatively low. None of the enrolled patients died.

**Fig. 2:**
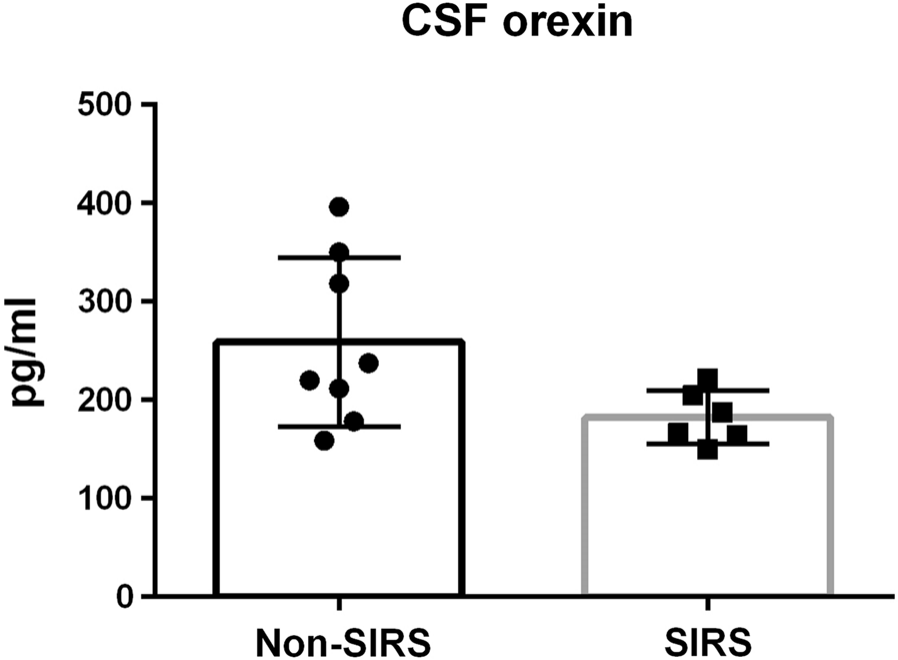
CSF orexin levels in SIRS patients were significantly lower than those in non-SIRS patients (non-SIRS: n = 8, 259.0 ± 85.7, SIRS: n = 6, 182.5 ± 27.2, F test: F = 9.9302, p = 0.0224, Welch’s test: p = 0.0424). Data are represented as the mean ± SD. *CSF* cerebrospinal fluid, *SIRS* systemic inflammatory response syndrome, *SD* standard deviation

We measured orexin levels in CSF and blood by using a standard orexin radioimmunoassay. The CSF orexin levels of the SIRS group were moderately low (< 200 pg/ml) and significantly lower than those of the non-SIRS group (Fig. 2). Blood orexin was detected in 3 patients. CSF orexin levels in patients with blood orexin detected were clinically low and significantly lower than those in patients with blood orexin undetected (Additional file 5: Fig. S2). Blood tests showed increased white blood cells and hyponatremia in patients with blood orexin detected (Additional file 3: Table S2).

### Discussion

In this study, we investigated orexin levels in the CSF and blood of rats and patients with systemic inflammation. The results for orexin levels in the CSF of rats with systemic inflammation were consistent with those for orexin levels in the CSF of patients with SIRS. The consistency between animal models and human patients is important to translate the results obtained from animal experiments to medical knowledge and/or novel therapies in humans, although we must carefully consider the differences between species. We expect that the results of this study can provide a novel understanding of the pathophysiology of sepsis and can be used as preliminary evidence to pave the way for a clinical trial of orexin in patients with sepsis.

In this patient study, we found that CSF orexin was low in SIRS patients with inflammation in the central nervous system (CNS). A previous study reported that CSF orexin was generally normal in patients with CNS inflammation but low in some patients [6]. Low CSF orexin levels in patients with CNS inflammation might depend on the occurrence of SIRS as a complication.

Additionally, we found that blood orexin could be detected in some patients with systemic inflammation and that CSF orexin levels were low in the patients with blood orexin detected. The findings were consistent with the observations in our previous case report [12], but not necessarily with the findings in rats with systemic inflammation. The inconsistency may depend on the differences in the structure and function of the blood–brain barrier between humans and rats. Again, in this human study, the abnormal detection of blood orexin could predict reduced CSF orexin levels. Interestingly, hyponatremia was observed in patients with blood orexin detected. These observations suggest that complications with syndrome of inappropriate secretion of antidiuretic hormone (SIADH) may occur. However, from the results of this study, it may be difficult to assess systemic inflammation based on blood orexin. Indeed, it has been reported that blood orexin does not reflect the severity of illness in intensive care unit patients with systemic inflammation [14].

## Limitations

This patient study was a single-center, retrospective observational study, and the sample size was small. We could not prospectively enroll patients with sepsis and sample CSF from them because of ethical issues. Instead, we retrospectively enrolled SIRS patients selected from those with CNS inflammation, and 83% of them were diagnosed with sepsis according to the previous definition based on SIRS. However, the signs and symptoms of enrolled SIRS patients were relatively mild and nonlethal, in contrast to the high mortality rate in sepsis. Therefore, the results of this patient study may be limited in their generalizability to all patients with sepsis.

While CNS dysfunction has recently been recognized to play a critical role in the pathophysiology of sepsis, the role of the CNS in patients with sepsis is not yet well characterized because of the difficulty of ethical brain research in patients with sepsis. Therefore, it may be important to accumulate the results obtained from rare cases or from studies that are feasible due to special conditions (such as this study), even if the sample sizes are small, to reveal the role of the CNS in patients with sepsis.

## Supplementary Information

**Additional file 1: Table S1.** Physical data and orexin levels in the included rats.

**Additional file 2: Figure S1.** In total, 1051 CSF and 1164 plasma samples were stored in the Department of Neurology, University of Tsukuba Hospital, from Jan 1, 2013, to Dec 31, 2018. Among them, 220 pairs of CSF and plasma samples were collected on the days of admission, of which 22 pairs were sampled from patients with meningitis/encephalitis. We ultimately enrolled 14 patients with meningitis/encephalitis because the CSF and plasma samples of 8 patients had already been used and were not available. CSF: cerebrospinal fluid.

**Additional file 3: Table S2.** Demographic, clinical data, and orexin levels in enrolled patients.

**Additional file 4: Table S3.** Comparison of blood tests between SIRS and non-SIRS patients.

**Additional file 5: Figure S2.** CSF orexin levels in patients with blood orexin detected were significantly lower than those in patients with blood orexin undetected (blood orexin (−): n = 11, 239.4 ± 81.2, ( +): n = 3, 177.5 ± 10.6, F test: F = 58.242, p = 0.03399, Welch test: p = 0.0318). *CSF* cerebrospinal fluid.

## Data Availability

The original data generated for the study are included in the article. Further inquiries may be directed to the corresponding author.

## Abbreviations

SIRS: Systemic inflammatory response syndrome
CSF: Cerebrospinal fluid
LPS: Lipopolysaccharide
qSOFA: Quick sequential organ failure assessment
CNS: Central nervous system
SIADH: Syndrome of inappropriate secretion of antidiuretic hormone
