# Supplementary figures and images for: Reduced CSF orexin levels in rats and patients with systemic inflammation: a preliminary study
Source: BMC Res Notes. 2022 Jun 25;15:221. doi: 10.1186/s13104-022-06121-0 (PMC9233848; doi:10.1186/s13104-022-06121-0)

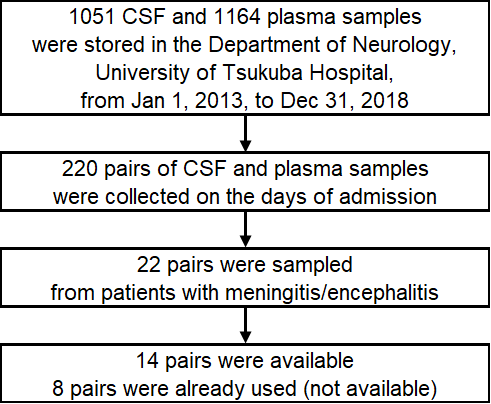

Supplement: Supplementary file 2 — Additional file 2: Figure S1. In total, 1051 CSF and 1164 plasma samples were stored in the Department of Neurology, University of Tsukuba Hospital, from Jan 1, 2013, to Dec 31, 2018. Among them, 220 pairs of CSF and plasma samples were collected on the days of admission, of which 22 pairs were sampled from patients with meningitis/encephalitis. We ultimately enrolled 14 patients with meningitis/encephalitis because the CSF and plasma samples of 8 patients had already been used and were not available. CSF: cerebrospinal fluid. [file 13104_2022_6121_MOESM2_ESM.png]

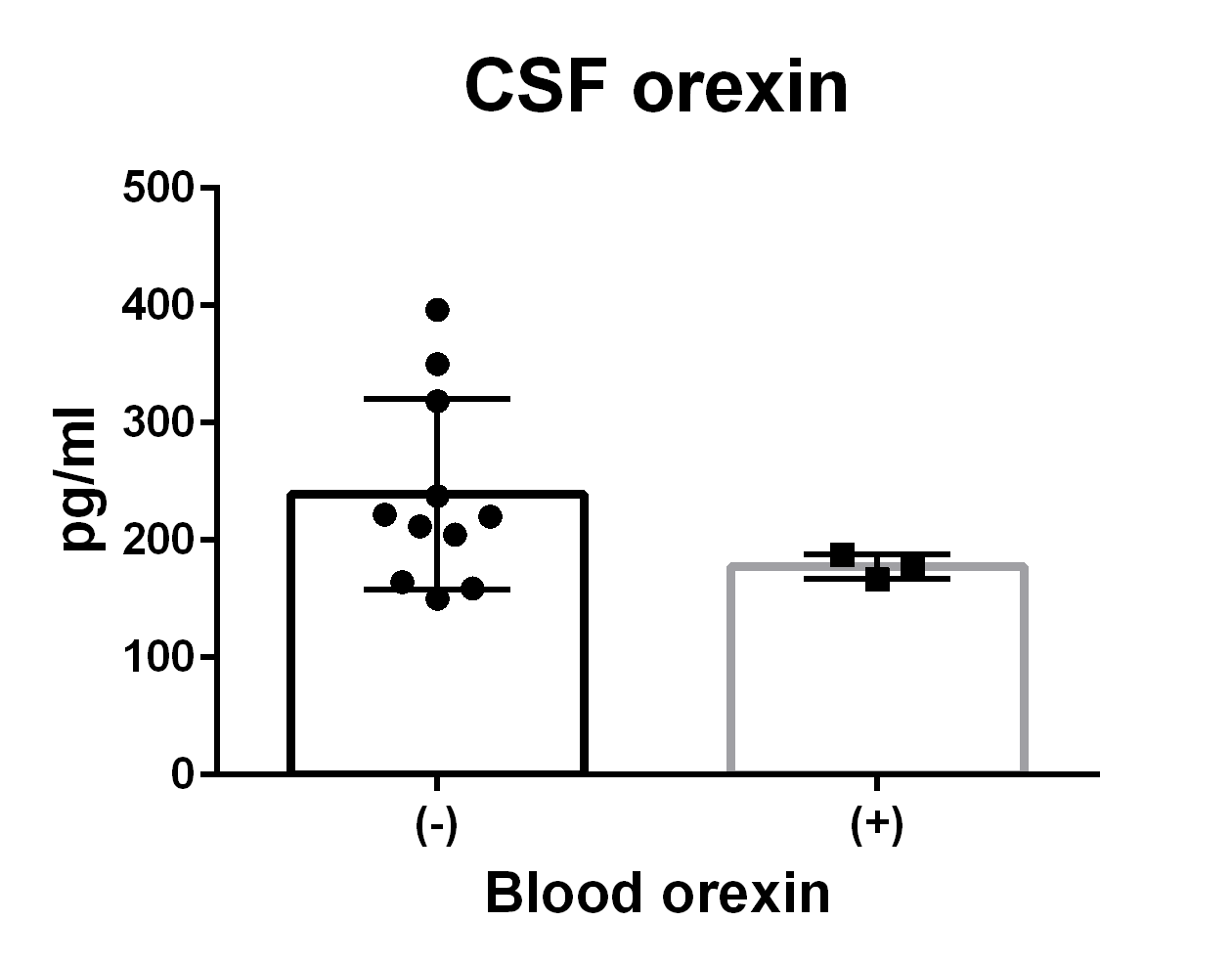

Supplement: Supplementary file 5 — Additional file 5: Figure S2. CSF orexin levels in patients with blood orexin detected were significantly lower than those in patients with blood orexin undetected (blood orexin (−): n = 11, 239.4 ± 81.2, ( +): n = 3, 177.5 ± 10.6, F test: F = 58.242, p = 0.03399, Welch test: p = 0.0318). CSF cerebrospinal fluid. [file 13104_2022_6121_MOESM5_ESM.png]
